# Designing Novel Antimicrobial Agents from the Synthetic Antimicrobial Peptide (Pep-38) to Combat Antibiotic Resistance

**DOI:** 10.3390/ph18060862

**Published:** 2025-06-10

**Authors:** Yara Al Tall, Yasmeen Alkurdi, Nid’A Alshraiedeh, Salsabeel H. Sabi

**Affiliations:** 1 Department of Pharmaceutical Technology, Faculty of Pharmacy, Jordan University of Science and Technology, Irbid 22110, Jordan; alkurdiyasmeen6@gmail.com (Y.A.); nhalshraiedeh@just.edu.jo (N.A.); 2 Basic Sciences Department, Faculty of Science, The Hashemite University, Zarqa 13133, Jordan; salsabeel@hu.edu.jo

**Keywords:** antimicrobial peptide, bioinformatics tools, bacterial susceptibility assay, antibiofilm assay, cytotoxicity assay, time–kill assay, PEP-38, Hel-4K-12K

## Abstract

**Background/Objectives:** The rise of antibiotic-resistant bacteria presents a major global health challenge, highlighting the need for novel antimicrobial agents such as antimicrobial peptides (AMPs). AMPs are promising due to their broad-spectrum activity, membrane-disruptive mechanisms, and low development of resistance. This study aimed to design and evaluate novel AMPs derived from a synthetic parent peptide (PEP-38). **Methods:** Novel peptides were designed using bioinformatics tools, including CAMP_R3_ and Peptide Ranker. Their antimicrobial potential was validated through in vitro assays, including bacterial susceptibility, antibiofilm activity, cytotoxicity, hemolysis, and time–kill kinetics. **Results:** Among the designed peptides, Hel-4K-12K showed potent activity against both Gram-positive and Gram-negative bacteria, with MICs ranging from 3.125 to 6.25 µM. It also effectively eradicated biofilms of resistant *Staphylococcus aureus* at an MBEC of 6.25 µM. Time–kill assays confirmed rapid bactericidal action, achieving complete bacterial elimination within one hour at its MIC. Moreover, Hel-4K-12K exhibited low toxicity toward mammalian MDCK cells (>82% viability at MIC) and minimal hemolytic activity on human erythrocytes. **Conclusions:** Hel-4K-12K demonstrates strong antibacterial and antibiofilm activities with a favorable safety profile, indicating its potential as a therapeutic candidate for treating infections caused by resistant bacteria. These findings support further development of this peptide as a basis for new antimicrobial drug strategies. In addition to its promising in vitro profile, future studies will investigate Hel-4K-12K in animal models and evaluate strategies for attaining stable formulations, such as peptide encapsulation or PEGylation. These steps are critical to ensure its therapeutic viability in systemic applications.

## 1. Introduction

The global health landscape is under increasing threat from the rise of antibiotic resistance, driven by the widespread and indiscriminate use of conventional antibiotics [1]. Multi-drug resistant bacteria (MDRB), which are resistant to multiple classes of antimicrobial agents, have become a major cause of morbidity and mortality worldwide, often resulting in treatment failures and escalating healthcare costs [2]. The Centers for Disease Control and Prevention (CDC) estimates that antimicrobial resistance (AMR) imposes an annual burden of approximately USD 55 billion in the USA, including USD 20 billion in direct healthcare costs and USD 35 billion in lost productivity [3]. Although AMR poses a global threat, its impact is particularly pronounced in low- and middle-income countries [4].

The emergence of MDR pathogens, particularly those in the ESKAPE group (*Enterococcus faecium*, *Staphylococcus aureus*, *Klebsiella pneumoniae*, *Acinetobacter baumannii*, *Pseudomonas aeruginosa*, and *Enterobacter* species), underscores the urgent need for innovative antimicrobial strategies [5]. Resistance can arise through various mechanisms, including intrinsic, adaptive, and acquired forms, with the latter posing the most immediate threat due to horizontal gene transfer [6].

The World Health Organization has listed AMR among the top ten global health threats [7], with pathogens such as carbapenem-resistant *Escherichia coli* and methicillin-resistant *Staphylococcus aureus* (MRSA) presenting significant challenges [8,9,10,11].

Antimicrobial peptides (AMPs) have emerged as promising alternatives to conventional antibiotics [12,13,14,15,16]. These short, cationic, and amphipathic molecules, naturally produced by most living organisms, exert rapid bactericidal effects through membrane disruption and intracellular targeting [17,18,19,20]. Unlike conventional antibiotics, AMPs tend to exhibit low host toxicity and a reduced propensity for resistance development, making them attractive candidates for next-generation anti-infective agents [21,22,23,24,25,26,27,28,29].

This study aimed to rationally design and evaluate novel AMPs derived from the synthetic helical peptide PEP-38, which previously demonstrated activity against carbapenem-resistant strains [30]. Through targeted amino acid substitutions, we sought to enhance PEP-38’s antimicrobial potency and selectivity while minimizing cytotoxicity. The modified peptides were assessed in vitro for antimicrobial, antibiofilm, and cytotoxic properties in comparison to the parent sequence. These findings support the potential of peptide-based therapeutics in addressing the urgent challenge of drug-resistant infections.

## 2. Results

### 2.1. Peptide Design, Molecular Modelling, and In Silico Analysis

#### 2.1.1. Result of CAMP_R3_ Analysis

The helical portion of the parent peptide sequence (PEP-38-Hel) (GLKDWVKKALGSLWKL) was investigated. The CAMP_R3_ website predicted an antimicrobial probability of 0.995 for this helical part. From the Rational Design of Antimicrobial Peptides tool of CAMP_R3_, 100 suggested peptide sequences for the helical peptide were obtained. The top five mutations with an antimicrobial probability of 0.999 were selected for further investigation (Table 1).

The modified peptides were designed accordingly based on CAMP_R3_’s top suggested mutations. Their characteristics were checked using bioinformatics tools.

#### 2.1.2. Results of NPS Secondary Structure Analysis

NPS HNN software was used to predict the helicity content of PEP-38 and the modified peptides. As shown in Table 2, all modified peptides’ helicity was increased compared with the parent peptide. The helical part of PEP-38 exhibited strong helicity, as expected. Replacing aspartic acid (D) at position 4 with arginine (R), lysine (K), and tryptophan (W) to a lesser degree (Hel-4R, Hel-4K, Hel-4W) maintained strong helicity. Replacing glycine (G) at position 11 or serine (S) at position 12 with lysine (K) (Hel-11K, Hel-12K) also enhanced helicity.

#### 2.1.3. Results Using the Innovagen Calculator

Innovagen Calculator software was used to calculate the net charge for the parent and the modified peptides, as shown in Table 2. The positive charge of AMPs is an important feature that leads to the electrostatic interaction of the peptides with the negatively charged bacterial membrane, with an optimum range from +2 to +9 according to the literature [31,32,33]. The charge of the modified peptides was lower than PEP-38 but rose when a positively charged amino acid, such as an arginine (R) (Hel-4R) or a lysine (K) (Hel-4K, Hel-11K, and Hel-12K), was added to the helical part.

#### 2.1.4. Results of ProtParam/ExPASY and APD3 Analysis

The physicochemical properties (aliphatic index, instability index, Boman index, GRAVY, and estimated half-life t_1/2_) of the parent and the modified peptides were calculated using the ProtParam/ExPASY server and the Antimicrobial Peptide Database (APD3) (Table 3). The instability index suggested that Hel-4R would be unstable in the test tube. At the same time, all the remaining sequences were stable with a value <40, and based on the Aliphatic index results, all the peptides can be regarded as thermostable. The GRAVY value assesses the hydropathy of the peptides; positive values indicate hydrophobicity and negative values indicate the hydrophilicity of the peptide. According to the results, all the peptides except Hel-4W were hydrophilic.

Building upon these findings, multiple substitutions were also tested in silico. As shown in Table 4, all the shortened peptides with an arginine (R) were unstable according to the instability index (Hel-4R-11K, Hel-4R-12K, and Hel-4R-11K-12K). On the other hand, all shortened peptides with tryptophan (W) showed lower helicity than others (Hel-4W-11K, Hel-4W-12K, and Hel-4W-11K-12K). Therefore, a decision was made to disregard these six peptides and focus on the shortened peptides with lysine (K) amino acids (Hel-4K-11K, Hel-4K-12K, Hel-11K-12K, and Hel-4K-11K-12K).

It is important to mention that all peptides had the same estimated half-life of 30 h in mammalian reticulocytes (in vitro), >20 h in yeast (in vivo), and >10 h in *E. coli* (in vivo). Additionally, all peptides had a Boman index value of less than one, indicating a lower potential for non-specific interactions with host proteins and potentially higher selectivity towards bacterial cells [34].

#### 2.1.5. Results of PeptideRanker

**Figure 1:**
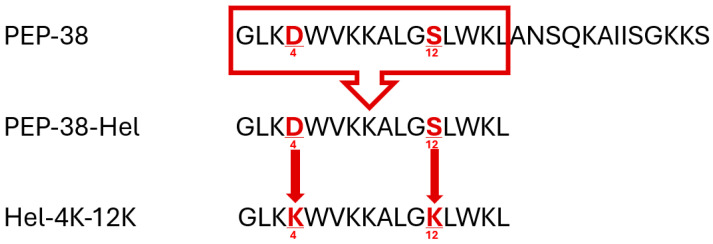
Visual mapping of peptide modifications from PEP-38 to Hel-4K-12K. The first 16 amino acids of PEP-38, used as the basis for derivative design, are boxed. PEP-38-Hel represents the truncated intermediate sequence. In Hel-4K-12K, residues at positions 4 and 12 (aspartic acid and serine) were substituted with lysine (K) to enhance cationicity and antimicrobial activity. Red bold characters highlight these key substitutions.

All the shortened peptides with lysine (K) demonstrated similar physicochemical properties based on ProtParam/ExPASY and APD3 analysis. To further prioritize the most promising candidates, the bioactivity of the shortlisted peptides (those containing lysine substitutions) was predicted using the PeptideRanker tool. According to Table 5, all tested peptides were expected to be bioactive, with PEP-38, PEP-38-Hel, and Hel-4K-12K exhibiting the highest probability scores. Consequently, these three peptides were selected for further investigation (Table 6; see also Figure 1 for a schematic comparison of their sequences).

### 2.2. Peptide Synthesis and Purification

The parent peptide (PEP-38), helical peptide (PEP-38-Hel), and Hel-4K-12K peptide were synthesized with purity >95% by the Canadian Biomatik company, using RP-HPLC. The identity of each peptide was verified using ESI-MS. The results are shown in the supplementary data (Supplementary S1–S3).

### 2.3. Bacterial Susceptibility Assay

The minimum inhibitory concentration (MIC) and minimum bactericidal concentration (MBC) of each of the peptides were determined using the micro broth dilution method against a panel of Gram-positive and Gram-negative bacteria, including *E. coli* (25922) and *S. aureus* (29213) as control strains, and *E. coli* (BAA-2452) and *S. aureus* (BAA-44) as multidrug-resistant strains. According to the MIC and MBC results in Table 7, the PEP-38-Hel peptide exhibited reduced antimicrobial activity against the control strains, compared with the parent peptide. Both peptides demonstrated similar activity against the resistant *S. aureus* strain, while the PEP-38-Hel was inactive against the resistant *E. coli* strain within the tested range of 0.78 µM to 100 µM (1.44 µg/mL to 184.22 µg/mL). On the other hand, the Hel-4K-12K peptide exhibited improved antimicrobial activity against all tested strains. All three peptides displayed bactericidal activity, as evidenced by MBC values equal to their MIC values.

### 2.4. Antibiofilm Activity Assay

The minimum biofilm eradication concentration (MBEC) method was used to assess the antibiofilm activities of the parent (PEP-38), PEP-38-Hel, and Hel-4K-12K peptides against *S. aureus* (ATCC BAA-44) and *E. coli* (ATCC 2452).

The results summarized in Table 8 demonstrate that all three peptides exhibited activity against *S. aureus* biofilms but not against *E. coli* biofilms. Notably, the Hel-4K-12K peptide displayed significantly enhanced antibiofilm activity compared with the parent peptide. These findings imply that specific peptide sequence mutations can significantly improve antibiofilm activity, making the Hel-4K-12K peptide a promising lead compound for developing novel antibiofilm agents.

### 2.5. Hemolytic Assay

**Figure 2:**
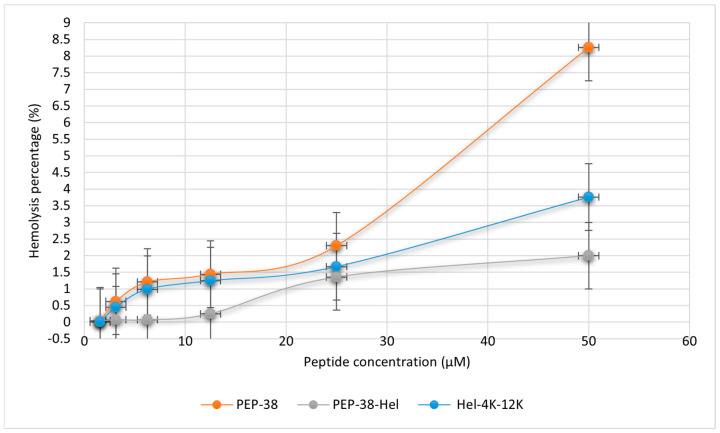
The hemolytic activity of PEP-38, PEP-38-Hel, and Hel-4K-12K at varying peptide concentrations (50, 25, 12.5, 6.25, and 3.125 μM).

The peptides’ possible toxicity to human red blood cells was assessed using the hemolytic assay. The safety of the peptides was confirmed by testing various concentrations against human red blood cells. Each peptide was incubated with a suspension of 4% human erythrocytes at concentrations of 50, 25, 12.5, 6.25, and 3.125 μM. The parent and modified peptides did not exhibit hemolytic activity at their respective MICs. The Hel-4K-12K peptide had minimal hemolytic activity even at 50 μM (16× MIC), indicating its excellent safety profile for potential therapeutic applications (Figure 2).

### 2.6. Mammalian Cell Cytotoxicity Assay

**Figure 3:**
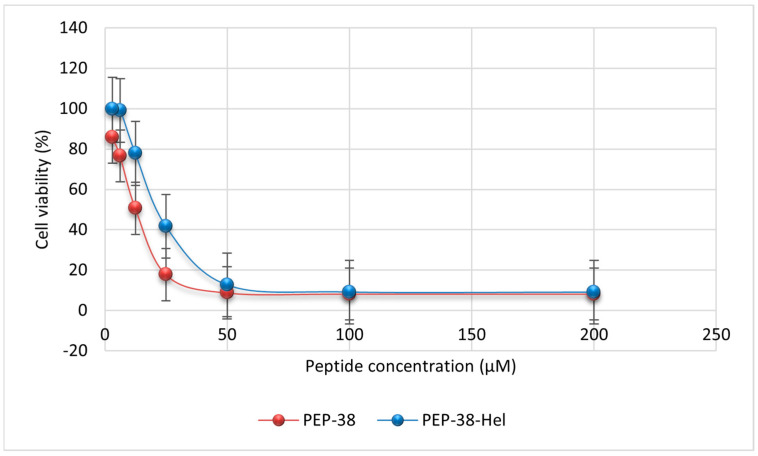
Cell viability of MDCK cells treated with increasing concentrations of the parent peptide (PEP-38) and the modified peptide (PEP-38-Hel), as determined by MTT assay.

**Figure 4:**
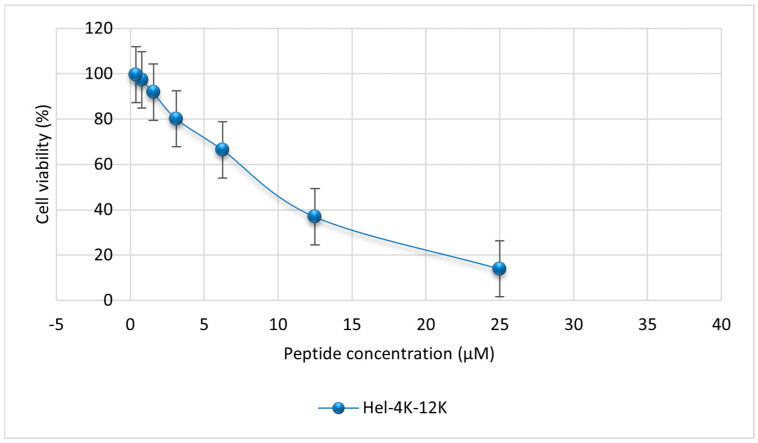
Cell viability of MDCK cells treated with increasing concentrations of the modified peptide (Hel-4K-12K), as determined by MTT assay.

The toxicity profiles of the parent PEP-38, PEP-38-Hel, and Hel-4K-12K peptides against MDCK cells were assessed using the MTT assay. Different concentrations of the parent (200, 100, 50, 25, 12.5, 6.25, and 3.125 μM), PEP-38-Hel (200, 100, 50, 25, 12.5, 6.25, and 3.125 μM), and Hel-4K-12K (25, 12.5, 6.25, 3.125, 1.5625, 0.78125, and 0.390625 μM) peptides were added to the MDCK cells. The cell viability was assessed based on the formation of formazan crystals, and the absorbances were quantified at λ = 570 nm using an ELISA microplate reader (EpochTM, BioTeck, Winooski, VT, USA). Figure 3 represents the cytotoxicities for PEP-38 and PEP-38-Hel peptides, and Figure 4 represents the cytotoxicity profile for the Hel-4K-12K peptide.

As shown in Table 9, the parent PEP-38 exhibited significant cytotoxicity at its MIC, particularly against both the control and resistant strains of *E. coli*. Although it was less toxic at the MIC needed to kill resistant *S. aureus*, its overall safety profile remained limited. Similarly, the PEP-38-Hel variant demonstrated a comparable toxicity pattern, with only slightly improved cytotoxicity against resistant *S. aureus*. In contrast, the Hel-4K-12K peptide exhibited a superior safety profile, maintaining over 82% cell viability at its MIC across all tested strains despite having a lower IC_50_ than PEP-38-Hel. This indicates that Hel-4K-12K can achieve effective antimicrobial activity without compromising mammalian cell viability at therapeutic concentrations.

### 2.7. Time–Kill Assay

**Figure 5:**
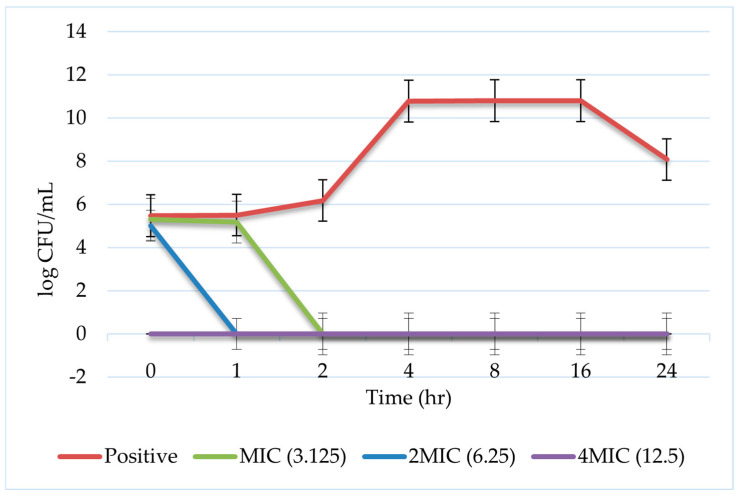
Time–kill kinetics of Hel-4K-12K peptide against *S. aureus* (BAA-44). Bacterial cultures were exposed to Hel-4K-12K for 0, 1, 2, 4, 8, 16, and 24 h. After the indicated incubation times, bacterial colonies were counted following an additional 24-h incubation period.

The time–kill assay curve for the Hel-4K-12K peptide against *S. aureus* (BAA-44) over a 24 h incubation time at 37 °C is represented in Figure 5.

In the time–kill kinetics assay, an immediate and significant reduction in bacterial growth was observed at 4× MIC (12.5 µM). Complete bacterial killing was observed at 2× MIC (6.25 µM) within one hour. At the MIC (3.125 µM), bacterial growth was initially unaffected during the first hour, followed by a rapid and significant decline at two hours. The rapid and sustained bactericidal activity of the Hel-4K-12K peptide, particularly at higher concentrations, highlights its potent antimicrobial efficacy.

## 3. Discussion

The use of in silico tools offers a streamlined, cost-effective strategy for AMP discovery, enabling rapid screening and design of peptides with enhanced antimicrobial properties and broad applicability [35,36].

This study employed bioinformatics platforms (e.g., CAMP_R3_) to optimize the synthetic peptide PEP-38, generating two analogs: PEP-38-Hel (helical segment) and Hel-4K-12K (with lysine substitutions at positions 4 and 12). In silico predictions suggested that increased helicity and net positive charge could enhance antimicrobial activity, consistent with Deslouches et al.’s findings on the helicity−activity correlation [37].

Among the designed peptides, Hel-4K-12K exhibited the most potent activity against both Gram-positive and Gram-negative bacteria. CAMP_R3_ scores confirmed its higher AMP probability, and in vitro results demonstrated up to 16-fold greater activity against *E. coli* and *S. aureus* compared to PEP-38-Hel. This highlights the role of residue positioning and cationic charge distribution in AMP efficacy [38,39].

Time–kill assays validated the rapid bactericidal effect of Hel-4K-12K against resistant *S. aureus*, achieving complete killing within one hour. This is consistent with AMPs’ known mechanism of action, targeting both bacterial membranes and intracellular components. Similarly, Zhang et al. (2024) demonstrated that the amphipathic peptide A24 rapidly disrupts the membrane integrity of multidrug-resistant *S. aureus*, leading to swift bacterial death [40].

Recent evidence from Matthyssen et al. (2024) also showed that dimerization and lysine substitution of melittin retained equivalent MIC and MBC values against *E. coli* and *S. aureus*, suggesting that such structural modifications preserve bactericidal mechanisms. These findings align with our observations for Hel-4K-12K and reinforce the principle that rational peptide design can enhance antimicrobial efficacy without altering mechanistic properties [41].

While increasing net positive charge is often linked to enhanced activity, this effect plateaus beyond a net charge of +8 due to toxicity risks [42]. Salazar-Hernández et al. (2024) showed that elevating peptide Uy234 net positive charge above +8 did not improve efficacy but significantly increased hemolytic activity [43]. In contrast, Hel-4K-12K, with a higher cationic charge than PEP-38-Hel, retained strong antimicrobial activity without inducing cytotoxicity, probably due to a favorable balance of charge, structure, and amphipathicity. This observation aligns with Gagat et al. (2024), who emphasized that selectivity depends on the balance between charge, hydrophobicity, and structure [44].

Biofilm disruption is critical for overcoming persistent infections. Hel-4K-12K displayed significant antibiofilm activity against resistant *S. aureus*, with MBEC values equal to or twice its MIC. However, no activity was observed against *E. coli* biofilms, contrasting with Kang et al.’s findings on Pseudin-2 but consistent with reduced efficacy of AMPs against Gram-negative biofilms [45,46]. This discrepancy may be attributed to the structural features of Gram-negative biofilm matrices, which contain negatively charged components such as alginate that neutralize cationic peptides [47]. In addition, the Gram-negative outer membrane of Gram-negative bacteria, which is rich in lipopolysaccharides, acts as a physical barrier, while efflux pumps contribute to intrinsic resistance by reducing intracellular peptide accumulation [48].

Toxicity profiling showed that Hel-4K-12K was the least cytotoxic peptide, with minimal hemolytic activity and over 82% cell viability at its MIC. Its moderate hydrophobicity and controlled helicity are likely to have contributed to this favorable profile. Although helicity and hydrophobicity have been linked to hemolysis [49,50], our results showed that PEP-38, despite being less helical, had higher hemolytic activity, indicating that other factors also contributed to cytotoxicity. Clark et al. (2021) supported this by showing that peptide length affects cytotoxicity [51].

Although lysine residues have been associated with hemolytic activity in some peptides [52], our findings challenge this generalization. Despite incorporating two lysine residues, Hel-4K-12K exhibited minimal hemolytic activity. This suggests that cytotoxicity depends on contextual factors such as sequence arrangement, charge distribution, and structural balance. Consistent with this, IC_50_ analysis revealed that Hel-4K-12K preserved higher mammalian cell viability than PEP-38 and PEP-38-Hel, supporting previous findings that rational design residue substitutions can reduce cytotoxicity.

Despite promising in vitro results, several limitations must be noted. First, all antimicrobial and antibiofilm assays were performed under controlled in vitro conditions, which may not fully reflect in vivo complexity [53]. Second, peptide stability in serum, particularly against proteases, was not assessed and this is a key factor for therapeutic application [54]. Third, while the design focused on helicity and charge, other physicochemical parameters like aggregation and intermolecular interactions were not fully explored [55]. Finally, the pathogen panel was limited; expanding it to include resistant clinical, multidrug-resistant isolates would enhance transitional relevance [56].

Collectively, our findings demonstrate that Hel-4K-12K is a potent, selective antimicrobial peptide. This study highlights the value of computational design to balance charge, helicity, hydrophobicity, and length for optimal efficacy and safety. Targeted amino acid substitutions, guided by bioinformatics tools, can generate peptides with improved therapeutic profiles.

Further studies are warranted to evaluate Hel-4K-12K in in vivo models and investigate its pharmacokinetics. Additionally, future work will assess the peptide stability in biologically relevant media such as serum and plasma, using degradation assays and incubation methods.

Although peptides were confirmed by the supplier using ESI-MS, this study primarily focused on antimicrobial and antibiofilm efficacy. Future research will incorporate physicochemical characterization to elucidate structure−activity relationships more thoroughly.

## 4. Materials and Methods

### 4.1. Materials and Bacterial Strains

The bacterial strains used in the present study were procured from the American Type Culture Collection (ATCC; Manassas, VA, USA) to assess the antibacterial activity of the parent, PEP-38-HEl, and Hel-4K-12K peptides (Table 10). Mueller Hinton Agar (Himedia Laboratories Pvt. Ltd., Mumbai, India) was used to cultivate the bacteria. The peptides were obtained from the Canadian Biomatik Corporation (Kitchener, ON, Canada, 2024), and the cell line used in the MTT assay was obtained from ATCC.

### 4.2. Methods

#### 4.2.1. Peptide Design and In Silico Analysis

Several bioinformatics tools were used to design novel peptides with appropriate physicochemical properties by modifying the helical part of the parent peptide PEP-38 (GLKDWVKKALGSLWKLANSQKAIISGKKS).

##### CAMP_R3_

The parent peptide sequence, PEP-38, was subjected to modifications using the Collection of Antimicrobial Peptides (**CAMP_R3_**) website [57]. The helical portion of PEP-38, encompassing the sequence GLKDWVKKALGSLWKL, exhibited predicted antimicrobial activity. Based on these predictions, **CAMP_R3_**’s rational design tool suggested potential mutations within this helical region. (https://camp.bicnirrh.res.in/, accessed on 5 June 2024).

##### NPS Secondary Structure Analysis

Hierarchical Neural Network in Network Protein Sequence (NPS HNN) software [58] was used to predict the helicity content of PEP-38 and its modified peptides. Helicity is a key determinant of peptide specificity and biological activity [59]. (https://npsa-prabi.ibcp.fr/cgi-bin/npsa_automat.pl?page=/NPSA/npsa_hnn.html, accessed on 5 June 2024).

##### Innovagen Calculator

The net charge of each peptide was calculated using PepCalc software version 49.3 [60]. This software calculates the net charge by summing the charges of individual amino acids within the peptide sequences. (https://pepcalc.com/, accessed on 5 June 2024).

##### ProtParam/ExPASy and APD3 Analysis

The ProtParam/ExPASy server (SIB Swiss Institute of Bioinformatics, Geneva, Switzerland) was used to predict the physical and chemical properties of the parent and modified peptides, including estimated half-life, instability index, aliphatic index, and grand average of hydropathy (GRAVY) [61]. The instability index reflects the peptide’s stability in a test tube according to the content of its amino acids, with values below 40 indicating stability [62]. The aliphatic index assesses a peptide’s thermostability based on the proportion of aliphatic amino acids (leucine, isoleucine, valine, and alanine) [63]. The grand average of hydropathy (GRAVY) index estimates a peptide’s hydrophobicity or hydrophilicity, with positive values indicating hydrophobicity and negative values indicating hydrophilicity [64]. (https://web.expasy.org/protparam/, accessed on 5 June 2024).

The Boman index, calculated using the APD3 website, predicts a peptide’s potential to bind to other proteins. Typically, Boman index values are negative or near zero [65]. (https://aps.unmc.edu/home, accessed on 5 June 2024).

##### PeptideRanker

The PeptideRanker server predicted the bioactivity of the modified peptides, as suggested by the **CAMP_R3_** rational design. This software ranks peptides based on their predicted bioactivity probability, ranging from 0 to 1, where higher scores indicate greater bioactivity potential [66,67]. (http://distilldeep.ucd.ie/PeptideRanker/, accessed on 6 June 2024).

#### 4.2.2. Peptide Synthesis and Purification

The peptides were produced in a lyophilized state using the solid-phase technique and Fmoc chemistry. Reverse-phase high-performance liquid chromatography (RP-HPLC) in an acetonitrile/H_2_O-TFA gradient was then used to investigate the peptides’ purity. The peptides’ identities were then verified using ESI-MS mass spectrometry.

#### 4.2.3. Bacterial Susceptibility Assay

Minimum inhibitory concentration (MIC) is the lowest concentration of an antimicrobial agent that will inhibit the visible growth of a microorganism after overnight incubation. The MIC is used to assess the potency of antimicrobial agents against pathogens and can guide selection of appropriate drug dosages [68].

Meanwhile, minimum bactericidal concentration (MBC) is the minimum concentration of the antimicrobial agent required to reduce more than 99.9% of the viability of the bacterial cells after incubating at 37 °C for 24 or 48 h, according to modified guidelines of the Clinical and Laboratory Standards Institute (CLSI) [69].

##### Determination of MIC and MBC of the Parent and the Modified Peptides

The antimicrobial activity of the parent, PEP-38-Hel, and Hel-4K-12K peptides was determined using the micro broth dilution method according to the guidelines provided by the CLSI [70].

Stock solutions containing 100 µM of peptide concentration were prepared by dissolving their powder in dimethyl sulfoxide (DMSO) and completing the volume with Muller Hinton broth (MHB) to obtain a solution of 5% DMSO in MHB. Serial dilutions were then performed in MHB to obtain eight different concentrations of each peptide: 100, 50, 25, 12.5, 6.25, 3.125, 1.56, and 0.78125 μM. Bacterial cells were cultured overnight on Muller Hinton agar, subsequently prepared with an initial concentration of 10^8^ CFU/mL, and then diluted 100-fold to reach 10^6^ CFU/mL. Equal volumes (50 µL) of the peptide concentrations and the bacterial suspension (10^6^ CFU/mL) were distributed into 96-well plates. A positive control was prepared by combining equal volumes (50 µL) of MHB and the bacterial suspension, while the negative control consisted of MHB alone. The plates were incubated at 37 °C for 18–24 h. Bacterial growth was then assessed by measuring the optical density (OD) at 600 nm using an enzyme-linked immunosorbent assay (ELISA) microplate reader (EpochTM; BioTek, Winooski, VT, USA).

The MBC of the peptides was determined by transferring 10 µL from turbidity-free wells of each peptide concentration, positive control wells, and negative control wells onto pre-sterilized MHB agar plates. The plates were then incubated at 37 °C for 24–48 h, following the CLSI guidelines. The MBC was determined by counting the number of bacterial colonies on each plate and comparing this with the initial count. The MBC is defined as the lowest concentration that results in a >99.9% reduction in bacterial viability compared with the initial count [71]. All MIC and MBC assays were made in triplicate.

#### 4.2.4. Hemolytic Assay

A hemolytic assay was used to evaluate the potential toxicity of the peptides on human cells and assess their hemolytic activity on human red blood cells.

Briefly, 48 mL of sterile phosphate-buffered saline (PBS) at PH 7.4 was combined with 2 mL of human blood (Sigma Aldrich, St. Louis, MO, USA). The suspension was then centrifuged three times for five minutes at 2000 rpm to separate red blood cells (RBCs) from the plasma. The RBC pellet was washed repeatedly with PBS to remove any remaining plasma or other cellular components. Finally, a 4% RBC suspension was prepared by resuspending the washed RBCs in PBS.

The hemolytic activity of five different concentrations of each peptide was determined using this 4% RBC suspension. A volume of 1 mL from each peptide concentration was added to 1 mL of 4% RBC suspension. The positive controls were prepared by mixing 2 mL of 4% RBC suspension with 5 μL of Triton 1×, and the negative controls consisted of 2 mL of 4% RBC suspension only. The five mixtures and the positive and negative controls were incubated at 37 °C for 1 h, followed by gentle vortexing of the tubes. After that, 500 μL of each sample supernatant was placed in pre-sterilized Eppendorf tubes and then centrifuged at 2000 rpm for 5 min. Then, 100 μL of each supernatant was placed in a well of the 96-well plate three times. The absorbance was measured using the ELISA reader at λ = 450 nm.

Next, the following equation was used to calculate the hemolysis rate:

(1) $$\mathrm{Hemolysis}\;\%=(\frac{A-\mathrm{AO}}{\mathrm{AX}-\mathrm{AO}})\;\times 100\%$$

where A represents the OD of the treated sample at λ = 450 nm, A0 is the OD of the negative control at λ = 450 nm, and AX is the OD of the positive control at λ = 450 nm.

#### 4.2.5. Mammalian Cell Cytotoxicity Assay

MDCK cells from Sigma-Aldrich (St. Louis, MO, USA), a kidney epithelial cell line isolated from a healthy adult female cocker spaniel, were cultured in RPMI 1640 media supplemented with L-glutamine, 1% *v*/*v* antibiotics (ampicillin-streptomycin), 1% *v*/*v* Amphotericin B as an antifungal agent, and 10% fetal bovine serum, all of which were purchased from Sigma-Aldrich (St. Louis, MO, USA). Cells were maintained at 37 °C in a humidified 5% CO_2_ incubator. The medium was replaced every 24 h until the confluency reached 70% to 80%. After discarding the medium, the adhering cells remained in the flask. Trypsin 1× was added in a volume of 3–5 mL, and the flask was then put back in the CO_2_ incubator for 15 min, tapping gently every 5 min.

Then, 5 mL of medium was added to counteract trypsin’s low pH, and centrifugation was run for 5 min at 2500 rpm. After discarding the supernatant, 10 mL of media was added and vortexed. We combined 50 μL of the cells with 50 μL of 4% trypan blue to stain the dead cells. Live unstained cells were counted using a hemocytometer.

Next, an MTT (3-(4,5-dimethylthiazolyl-2)-2, 5-diphenyltetrazolium bromide) assay was performed to assess cell viability. MDCK cells were seeded at a density of 5 × 10^3^ cells/well on a 96-well plate. The plates were incubated overnight at 37 °C in a CO_2_ incubator to reattach the cells. After removing the media from the plates, cells were treated with various concentrations of parent (PEP-38), PEP-38-Hel, and Hel-4K-12K peptides.

Following a 24-h incubation at 37 °C in a CO_2_ incubator, 25 µL of MTT solution (4 mg/mL) was added to each well. Mitochondrial enzymes in viable cells reduced the yellow MTT dye to purple formazan crystals. After 4–6 h, the formazan crystals were dissolved in 100 µL of DMSO, and the absorbance was measured at 570 nm using an ELISA microplate reader (EpochTM, BioTeck, Winooski, VT, USA). The assay was made in duplicate for each peptide.

#### 4.2.6. Antibiofilm Activity Assay

##### Biofilm Formation

Resistant strains of *E. coli* (ATCC 2452) and *S. aureus* (ATCC BAA-44) were used to investigate the peptides’ antibiofilm activities. A 10^7^ CFU/mL bacterial suspension was prepared, and 150 μL was inoculated into each well of 96-well plates. The negative controls included 150 μL of MHB. Plastic pegs were used to facilitate biofilm formation. The plates were incubated at 37 °C with shaking at 110 rpm for 14–16 h.

##### Challenge Plate

The biofilm-coated pegs were washed three times with 200 μL sterile PBS to eliminate planktonic cells. The pegs were then transferred to new 96-well plates containing 200 μL of various peptide concentrations, from 0.39 μM to 200 μM for the PEP-38 and PEP-38-Hel peptides and from 0.2 μM to 100 μM for the Hel-4K-12K peptide. The challenge plates were incubated at 37 °C with shaking at 110 rpm for 24 h.

##### Recovery Plate

The pegs were washed three times with 200 μL PBS and placed in a clean 96-well plate with 200 μL PBS/well. The biofilm cells were detached from the pegs by sonication for 20 min. After that, 50 μL of biofilm suspension from each well was transferred to new plates containing 50 μL of fresh MHB. The plates were incubated at 37 °C for 18 h. The minimum biofilm eradication concentration (MBEC) was measured at 595 nm using an ELISA reader.

#### 4.2.7. Time–Kill Assay

The Hel-4K-12K peptide was evaluated against *S. aureus* BAA-44 in a time–kill assay. Peptide solutions were prepared at MIC, 2× MIC, and 4× MIC concentrations. A 96-well plate was filled with 50 μL of each peptide concentration and 50 μL of a 10^6^ CFU/mL BAA-44 bacterial suspension. Positive controls contained bacterial suspension, while negative controls contained only MHB.

At 0, 1, 2, 4, 8, 16, and 24 h, 10 μL aliquots were transferred from each well to a new 96-well plate containing 90 μL of PBS. Then, 10 μL from each well was then plated onto nutrient agar plates. The plates were incubated at 37 °C for 24 h. Colony-forming units (CFUs) were counted, and the bacterial killing rate was determined by calculating the log reduction in CFU/mL compared with the initial inoculum.

## 5. Conclusions

In conclusion, the Hel-4K-12K peptide demonstrated potent antimicrobial activity against both control and resistant strains of *E. coli* and *S. aureus*, along with significant antibiofilm activity against resistant *S. aureus*. It also exhibited no hemolytic activity and minimal cytotoxic effects on mammalian cells, supporting its potential as a therapeutic candidate for combating multidrug-resistant infections.

This study highlights the effectiveness of rational design modifications to the helical region of PEP-38 in enhancing antimicrobial potency while maintaining a favorable safety profile. While the current in vitro findings are promising, in vivo validation in animal infection models is essential to assess this peptide’s pharmacokinetics, therapeutic efficacy, and systemic safety.

Further efforts will also explore formulation strategies such as liposomal encapsulation, hydrogel-based delivery, and PEGylation to improve the peptide’s stability, extend its circulation time, and facilitate targeted delivery. These approaches aim to support the translational development of Hel-4K-12K for clinical applications.

## Figures and Tables

**Table 1:** Antimicrobial peptide prediction for PEP-38 and derivatives (Hel: Helical part) using the support vector machine (SVM) classifier from CAMP_R3_.

Peptide ID.	Position	Sequence
Hel-4R	4-R	GLK**R**WVKKALGSLWKL
Hel-4K	4-K	GLK**K**WVKKALGSLWKL
Hel-4W	4-W	GLK**W**WVKKALGSLWKL
Hel-11K	11-K	GLKDWVKKAL**K**SLWKL
Hel-12K	12-K	GLKDWVKKALG**K**LWKL

**Table 2:** Comparison of helicity predictions, amino acid counts (a.a. no.), and net charge for PEP-38 and modified peptides (NPS HNN software and Innovagen Calculator).

Peptide ID.	Peptide Sequence	a.a. no.	Helicity	Net Charge
PEP-38	**GLKDWVKKALGSLWKL**ANSQKAIISGKKS	29	51.72%	+6
PEP-38-Hel	**GLKDWVKKALGSLWKL**	16	81.25%	+3
Hel-4R	GLK**R**WVKKALGSLWKL	16	81.25%	+5
Hel-4W	GLK**W**WVKKALGSLWKL	16	68.75%	+4
Hel-4K	GLK**K**WVKKALGSLWKL	16	81.25%	+5
Hel-11K	GLKDWVKKAL**K**SLWKL	16	81.25%	+4
Hel-12K	GLKDWVKKALG**K**LWKL	16	81.25%	+4

**Table 3:** Bioinformatics analysis of PEP-38 and its CAMP_R3_-designed modified peptides, ProtParam/ExPASY and APD3 results.

Peptide ID.	Peptide Sequence	Instability Index	Aliphatic Index	GRAVY	Boman Index (Kcal/mol)
PEP-38	G**LKDWVKKALGSLWKL**ANSQKAIISGKKS	11.76	101.3	−0.352	0.92
PEP-38-Hel	G**LKDWVKKALGSLWKL**	6.46	121.88	−0.081	0.14
Hel-4R	GLK**R**WVKKALGSLWKL	62.63	121.88	−0.144	0.52
Hel-4W	GLK**W**WVKKALGSLWKL	6.46	121.88	0.081	**−0.55**
Hel-4K	GLK**K**WVKKALGSLWKL	6.46	121.88	−0.106	−0.05
Hel-11K	GLKDWVKKAL**K**SLWKL	1.15	121.88	−0.300	0.54
Hel-12K	GLKDWVKKALG**K**LWKL	−4.16	121.88	−0.275	0.27

**Table 4:** A comparison of in silico predicted physicochemical properties of PEP-38 and its modified variants with substitutions at positions 4, 11, and 12. Properties include peptide sequences, amino acid count (a.a. no.), helicity, net charge, and instability index.

Peptide ID.	Peptide Sequence	a.a no.	Helicity	Net Charge	Instability Index	Aliphatic Index	GRAVY	Boman IndexKcal/mol
**Hel-4R-11K**	GLK**R**WVKKAL**K**SLWKL	16	81.25%	+6	**57.33**	121.88	−0.362	0.93
**Hel-4R-12K**	GLK**R**WVKKALG**K**LWKL	16	81.25%	+6	**52.02**	121.88	−0.338	0.66
**Hel-4R-11K-12K**	GLK**R**WVKKAL**KK**LWKL	16	81.25%	+7	**52.02**	121.88	−0.556	1.06
**Hel-4W-11K**	GLK**W**WVKKAL**K**SLWKL	16	**68.75%**	+5	1.15	121.88	−0.138	−0.14
**Hel-4W-12K**	GLK**W**WVKKALG**K**LWKL	16	**68.75%**	+5	−4.16	121.88	−0.113	−0.41
**Hel-4W-11K-12K**	GLK**W**WVKKAL**KK**LWKL	16	**68.75%**	+6	−4.16	121.88	−0.331	−0.01
Hel-4K-11K	GLK**K**WVKKAL**K**SLWKL	16	81.25%	+6	1.15	121.88	−0.325	**0.34**
Hel-4K-12K	GLK**K**WVKKALG**K**LWKL	16	81.25%	+6	−4.16	121.88	−0.300	**0.07**
Hel-11K-12K	GLKDWVKKAL**KK**LWKL	16	81.25%	+5	−4.16	121.88	−0.494	**0.68**
Hel-4K-11K-12K	GLK**K**WVKKAL**KK**LWKL	16	81.25%	+7	−4.16	121.88	−0.519	**0.48**

**Table 5:** Predicted bioactivity scores of the peptides as determined by PeptideRanker.

Substitution Position	Probability	Peptide Sequence
PEP-38	0.917525	GLKDWVKKALGSLWKLANSQKAIISGKKS
PEP-38-Hel	0.912204	GLKDWVKKALGSLWKL
Hel-4K-12K	0.910295	GLK**K**WVKKALG**K**LWKL
Hel-11K-12K	0.904663	GLKDWVKKAL**KK**LWKL
Hel-4K-11K-12K	0.904145	GLK**K**WVKKAL**KK**LWKL
Hel-4K-11K	0.899625	GLK**K**WVKKAL**K**SLWKL

**Table 6:** Finalized peptide sequences for further analysis, including their amino acid count (a.a. no.) and molecular weight (M.W.) calculated using the ProtParam/ExPASY server.

Peptide Name	Sequence	a.a.no.	M.W. (g/mol)
PEP-38	GLKDWVKKALGSLWKLANSQKAIISGKKS	29	3155.73
PEP-38-Hel	GLKDWVKKALGSLWKL	16	1842.23
Hel-4K-12K	GLK**K**WVKKALG**K**LWKL	16	1896.41

**Table 7:** Minimum inhibitory concentration (MIC) and Minimum bactericidal concentration (MBC) for PEP-38, PEP-38-Hel, and Hel-4K-12K peptides against control and resistant strains of *Escherichia coli* (*E. coli)* and *Staphylococcus aureus (S. aureus)*.

Bacterial Strain	ATCC Number	PEP-38		PEP-38-Hel		Hel-4K-12K	
		MIC	MBC	MIC	MBC	MIC	MBC
***E. coli***	25922	25 µM	25 µM	50 µM	50 µM	3.125 µM	3.125 µM
***E. coli***	BAA-2452	25 µM	25 µM	NA ^1^	NA ^1^	6.25 µM	6.25 µM
***S. aureus***	29213	25 µM	25 µM	50 µM	50 µM	3.125 µM	3.125 µM
***S. aureus***	BAA-44	12.5 µM	12.5µM	12.5 µM	12.5 µM	3.125 µM	3.125 µM

^1^ Not Active.

**Table 8:** Minimum biofilm eradication concentration (MBEC) of the peptides for eradicating biofilms of a resistant strain of *Staphylococcus aureus* (BAA-44).

Bacterial Strain	Peptide ID.	MBEC (μM)
*Staphylococcus aureus* **(ATCC BAA-44)**	PEP-38	12.5
	PEP-38-Hel	25
	Hel-4K-12K	6.25

**Table 9:** Half-maximal inhibitory concentration (IC₅₀) of peptides determined by MTT assay for cell viability.

Peptide ID.	IC_50_ (μM)
PEP-38	13.25
PEP-38-Hel	24.89
Hel-4K-12K	7.96

**Table 10:** Bacterial strains used in this study and their characteristics.

ATCC Number	Bacterial Strain	Characteristics
25922	*Escherichia coli* (*E. coli*)	Control strain
BAA-2452	*Escherichia coli* (*E. coli*)	Resistant strain
29213	*Staphylococcus aureus (S. aureus)*	Control strain
BAA-44	*Staphylococcus aureus (S. aureus)*	Resistant strain

## Data Availability

Data are contained within the article and Supplementary Materials.

## Supplementary Materials

The following supporting information can be downloaded at: https://www.mdpi.com/article/10.3390/ph18060862/s1, Supplementary S1: Certificate of Analysis for PEP-38, including High-performance liquid chromatography (HPLC) chromatogram and Mass spectrometry (MS) analysis; Supplementary S2: Certificate of Analysis for PEP-38-Hel peptide including High-performance liquid chromatography (HPLC) chromatogram and Mass spectrometry (MS) analysis; Supplementary S3: Certificate of Analysis for Hel-4K-12K peptide including High-performance liquid chromatography (HPLC) chromatogram and Mass spectrometry (MS) analysis.

## Abbreviations

The following abbreviations are used in this manuscript:

Λ	Lambda
µL	Micro-liter
µM	Micro-molar
AI	Aliphatic index
AMR	Antimicrobial resistance
AMPs	Antimicrobial peptides
ATCC	American Type Culture Collection
CAMP_R3_	Collection of Antimicrobial Peptides
CFU	Colony-forming unit
CLSI	Clinical and Laboratory Standards Institute
DMSO	Dimethyl sulfoxide
EDTA	Ethylenediaminetetraacetic acid
ELISA	Enzyme-linked immunosorbent assay
EPS	Extracellular polymeric substance
EU	European Union
GRAVY	Grand average of hydropathy
HDP	Host defense peptide
HEL	Helical
HPLC	High-performance liquid chromatography
LA	Lipoteichoic acid
LSTM	Long short-term memory
MBC	Minimum bactericidal concentration
MBEC	Minimum biofilm eradication concentration
MDRB	Multi-drug-resistant bacteria
MHB	Mueller Hinton broth
MIC	Minimum inhibitory concentration
MRSA	Multidrug-resistant *Staphylococcus aureus*.
MS	Mass spectrometry
MTT	Dimethyl thiazolyl diphenyl tetrazolium
NPS	Network protein sequence
OD	Optical density
PBS	Phosphate buffer saline
RBC	Red blood cell
RF	Random forest
RNN	Recurrent neural network
Rpm	Rounds per minute
SVM	Support vector machine
UK	United Kingdom
US	United States
WHO	World Health Organization
