# Supplementary material for: Designing Novel Antimicrobial Agents from the Synthetic Antimicrobial Peptide (Pep-38) to Combat Antibiotic Resistance
Source: Pharmaceuticals (Basel). 2025 Jun 10;18(6):862. doi: 10.3390/ph18060862 (PMC12195976; doi:10.3390/ph18060862)
Supplement: Supplementary file 1 [file pharmaceuticals-18-00862-s001.zip › Supplementary S3.pdf]

Biomatik
Tel: (519) 489-7195, (800) 836-8089
Fax: (519) 231-0140, (877) 221-3515
Email: info@biomatik.com
http://www.biomatik.com

Certificate of Analysis

**Date:**	2024-07-03
**Order Number:**	#SP240743
**Product Type:**	Chemically synthesized peptide
**Catalog Number:**	1177201
**Peptide Name:**	HEL-4K-12K
**Sequence (N to C):**	GLKKWVKKALGKLWKL
**MW:**	1896.41
**Salt Form:**	Trifluoroacetate (TFA Salt)
**Quantity:**	50.0mg
**Suggested Solvent:**	1.0mg peptide soluble in 1.0ml ($H2O$:Acetonitrile=4:1)
**Lot Number:**	P240617-LR1177201
**Appearance:**	White to off-white lyophilized powder.
**Storage:**	Store lyophilized peptide at -20°C upon receipt. Reconstitute only the amount of peptide needed for immediate use.
**Limited Usage:**	For Research Use Only. Not for use in diagnostic procedures, or for administration to humans or animals.

ASSAY	SPECIFICATION	ACTUAL
**MW by MS**	1896.30	Conforms
**Purity by HPLC**	>95%	95.31%
**Peptide Content**	N/A	N/A
**TFA Content**	N/A	N/A
**Moisture Content**	N/A	N/A

Biomatik
Tel: (519) 489-7195, (800) 836-8089
Fax: (519) 231-0140, (877) 221-3515
Email: info@biomatik.com
http://www.biomatik.com

MS REPORT

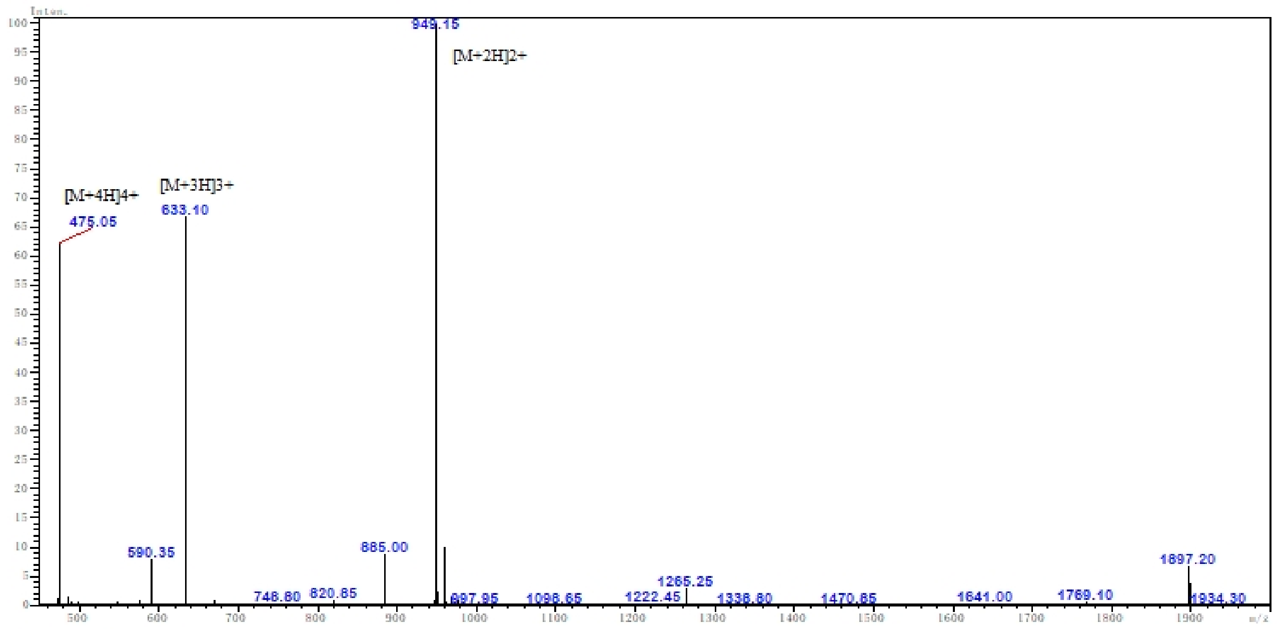

Acquired by : Huang
Data Acquired : 2024/7/1
Injection Volume : 1
Sample Name :HEL-4K-12K GL-16
Mw :1896.41
Lot No. :P240617-LR1177201

Probe	:ESI	Probe bias	:+4.5kv
Nebulizer Gas Floe	:1.5L/min	Detector	:1.2kv
CDL	:-20.0v	T.Flow	:0.2ml/min
CDL Temp	:250℃	B.conc	:50%H2O/50%ACN
Block Temp	:400℃		

Biomatik
Tel: (519) 489-7195, (800) 836-8089
Fax: (519) 231-0140, (877) 221-3515
Email: info@biomatik.com
http://www.biomatik.com

HPLC REPORT

Structure :HEL-4K-12K GL-16
Lot NO :P240617-LR1177201
Number :0200049
Column :4.6×250mm,ChromCore 120 C18 5u
Solvent A:0.1%TFA in 100%water
Solvent B:0.1%TFA in 100%acetonitrile

Gradient :	A	B
0.1min	71%	29%
25.0min	46%	54%
25.1min	0%	100%
30.0min	stop	

Flow rate:1.0ml/min
Wavelength(nm):220
Volume :10ul

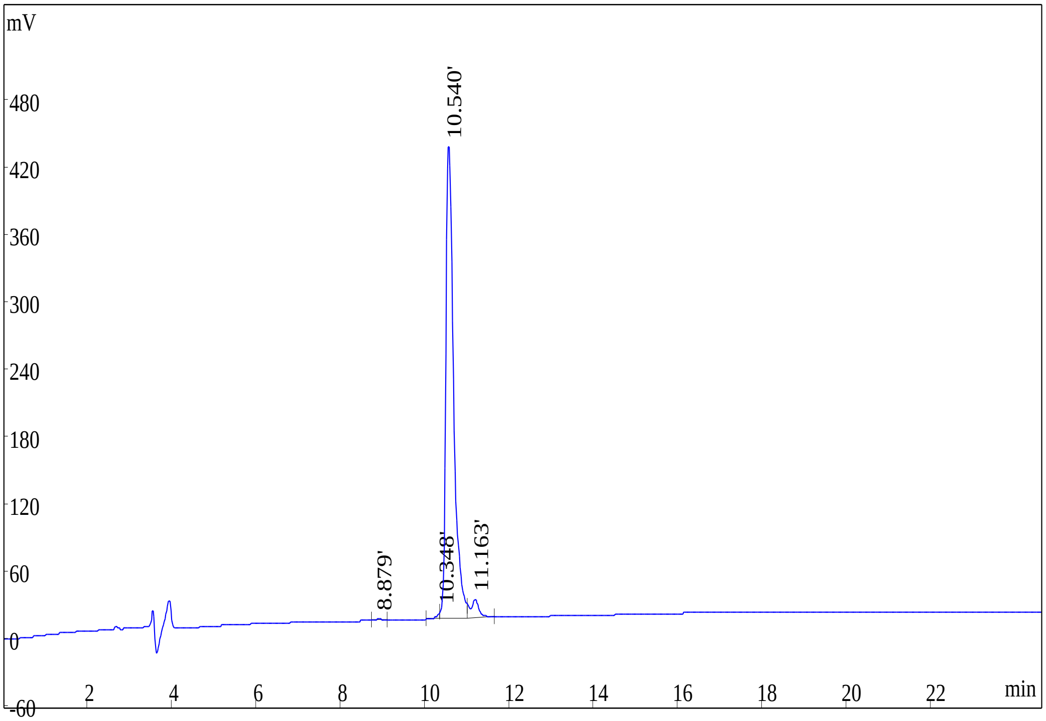

Rank	Time	Conc.	Area	Height
1	8.879	0.2272	11929	1249
2	10.348	0.5747	30180	6045
3	10.540	95.31	5004869	419774
4	11.163	3.89	204279	16797
Total		100	5251257	443865
